# Tribological Properties of Single (AlSi7/SiC_p,_ AlSi7/GC_sf_) and Hybrid (AlSi7/SiC_p_ + GC_sf_) Composite Layers Formed in Sleeves via Centrifugal Casting

**DOI:** 10.3390/ma12172803

**Published:** 2019-08-30

**Authors:** Anna Janina Dolata, Maciej Dyzia, Jakub Wieczorek

**Affiliations:** Faculty of Materials Engineering and Metallurgy, Silesian University of Technology, Krasińskiego 8, 40-019 Katowice, Poland

**Keywords:** metal matrix composites, hybrid reinforcement, centrifugal casting, composite sleeve, tribological properties

## Abstract

When designing the composition and structure of a composite material intended for tribological cooperation, many external and structural factors must be considered. The aim of this research was to compare the tribological properties (wear resistance and friction coefficient) of AlSi7Mg1Sr0.03/SiC_p_ and AlSi7Mg1Sr0.03/GC_sf_ single-reinforced composite layers with AlSi7Mg1Sr0.03/SiC_p_ + GC_sf_ hybrid composite layer formed in sleeves via vertical centrifugal casting. Profilometry enabled quantitative and qualitative analyses to be performed on the wear traces formed on investigated surfaces. The results show that a hybrid composite layer containing spherical glassy carbon particles had a significantly lower and more stable coefficient of friction (μ) and a higher wear resistance compared with single composite layers. The obtained effect was related to the mechanism of vitreous carbon consumption, which was crushed during operation, and then introduced between the cooperating friction surfaces. In this way, it acted as a solid lubricant, which stabilized the coefficient of friction and reduced the wear process.

## 1. Introduction

The largest group of metal matrix composites (MMCs) are based on aluminum-silicon alloy matrixes (AlMCs), mainly due to their favorable prices and properties, particularly their low density (~2.7 g/cm^3^), high strength/weight ratio, corrosion resistance, and their ability to be formed and treated. Additionally, the introduction of various ceramic components into aluminum alloys allows their properties to be tailored to a wide range of engineering applications [1]. For example, the introduction of hard Al_2_O_3_ or SiC ceramic phases to an AlSi alloy allows the production of materials with enhanced wear resistance under friction [2,3,4,5,6,7]. The introduction of graphite particles provides good sliding properties [8,9]. In turn, the use of different dispersed phases (i.e., TiC, SiC, Al_2_O_3_, or intermetallic phases) increases the yield point and creep resistance, making it possible to use materials at elevated temperatures [10,11,12]. Sajjadi et al. [13] and many others have reported improvements in strength and wear resistance by reducing the reinforcement size from the micro to the nano scale [14,15,16]. These developments are interesting, but some problems related to the manufacture of nanocomposites, as well as high costs and availability of nano-components, limit their commercialization. 

Recently, there has been an increasing number of research works related to the development of hybrid aluminum matrix composites (HAlMCs), which contain two or more ceramic components with different properties [17,18,19,20,21,22,23,24,25,26,27,28]. The main advantage of using hybrid-reinforced materials compared with composites with only one particle type is the possibility of achieving synergistic strengthening and lubrication effects [26,27,28]. In the literature, various methods have been described for the production of aluminum-based hybrid composites. Similar to homogenous reinforced composites, HAlMCs are generally produced via two routes: powder metallurgy (solid-state) or melting metallurgy processes (liquid-state). The solid-state route involves powder metallurgy techniques, i.e. mechanical alloying followed by hot pressing, while the liquid-state route is based mainly on stir casting techniques. The next step is to shape the finished products via high-pressure die casting, squeeze casting, or centrifugal casting. Compared with the unit methods based on powder metallurgy proposed and described by Hekner et al. [26,27], centrifugal casting methods are similar to squeeze casting [29], making it possible to obtain a series of near-net shape castings that are also locally reinforced [30]. An example of a practical use of such solutions are locally-reinforced pistons and engine blocks obtained by the high-pressure infiltration of ceramic preforms using liquid Al alloys [31,32]. Using a preform with 12% volume fraction of Al_2_O_3_ short fibers and 9% carbon fibers decreased the weight of the engine block, improved cooling, and reduced friction wear. However, it should be noted that high-pressure methods are more expensive than casting methods, especially when used for single-unit and short-run production.

Therefore, many scientists continue to research the development of efficient and economical manufacturing processes for various machine parts with local ceramic reinforcements. Such solutions significantly reduce the use of demanding and costly machining process of metal-ceramic composite finished products [33,34,35]. Sobczak and Drenchev described various methods to obtain composite materials with non-uniform, functional gradients or layered distributions of reinforcement materials in an aluminum matrix [36]. Apart from complex and costly techniques based on high-pressure casting processes, centrifugal casting methods [37,38,39,40] have great potential for wider commercialization and for the production of various automotive machine parts [41,42]. For example, hypereutectic aluminum alloy-based composite pistons partially reinforced at the head with SiC particles obtained by centrifugal casting have been successfully manufactured by Huang et al. [43].

In turn, studies conducted by Rajan and Pai [42] showed that centrifugal casting can be successfully used to fabricate lightweight hybrid composite gear wheels, cylinder liners, and brake rotor discs. They demonstrated that in the case of a hollow cylinder, a specific gradation of SiC and graphite particles towards the inner periphery could be used to create a hybrid, functionally graded composite. Many studies on the wear properties of this kind of hybrid composites have clearly demonstrated that the presence of graphite particles has a smaller influence on the direct reduction of abrasive wear in terms of material mass loss. However, it plays an important role as a lubricant for working surfaces of the matrix and the SiC particles themselves [44,45].

Our research focuses on the possibility of using glassy carbon (GC_p_) as an alternative lubricant to graphite particles (GR_p_). In one of the first studies described in [46], 5 wt.% (GC_p_) lamellar particles were used to modify the tribological properties of AlSi12CuNiMg/25 wt.% Al_2_O_3_ and AlSi12CuNiMg/25 wt.% SiC_p_ composite sleeves produced by centrifugal casting at speed of 500 rpm. The obtained results showed that the addition of glassy carbon, irrespective of the type of ceramic reinforcement, changed the tribological properties of the system. It also contributed to the stabilization of the friction coefficient as a function of friction distance and almost completely eliminated the grinding-in period of the cooperating partners. Moreover, a decrease in the friction coefficient in all hybrid composites was observed.

The aim of this research was to compare the tribological properties (wear resistance and friction coefficient) of AlSi7Mg1Sr0.03/SiC_p_ and AlSi7Mg1Sr0.03/GC_sf_ single-reinforced composite sleeves with AlSi7Mg1Sr0.03/SiC_p_ + GC_sf_ hybrid composite obtained via vertical centrifugal casting at speed of 3000 rpm. Compared with previous works, a much lower reinforcing particle content was used in both the single and hybrid composites. In addition, spherical glassy carbon and a matrix with a lower Si content were used to reduce the brittleness of the composite material. Moreover, profilometric studies were carried out to quantitatively and qualitatively analyze the wear traces formed on the investigated surfaces.

## 2. Materials and Methods

**Figure 1:**
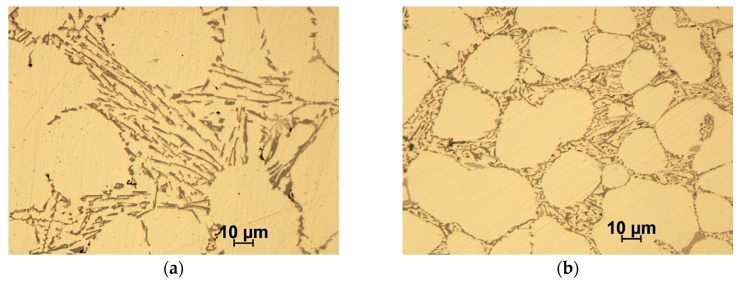
Microstructure of EN AC 42200 (AlSi7Mg0.6), OM: (**a**) base alloy; (**b**) base alloy after modification by Mg and Sr.

The EN AC 42200 (AlSi7Mg0.6) commercial alloy with a known chemical composition (Table 1) was used as the base alloy to prepare the composite matrix (Figure 1a). To improve the wetting conditions in the ceramic-metal system [47], the composition of the base alloy was modified by adding Mg and Sr (Figure 1b). For this purpose, AlMg25 and AlSr10 master alloys produced by the Institute of Non-Ferrous Metals in Skawina were used.

**Figure 2:**
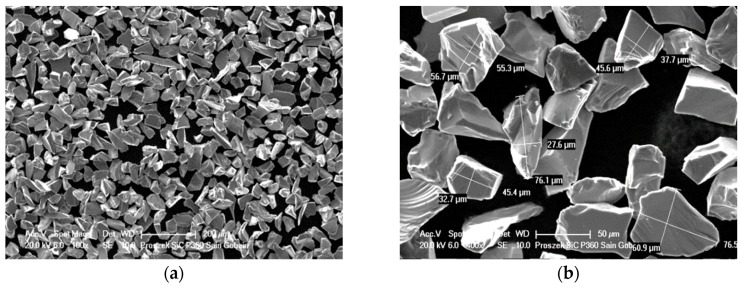
Scanning electron microscopy (SEM) images showing the morphology of irregular silicon carbide particles (SiC_p_).

**Figure 3:**
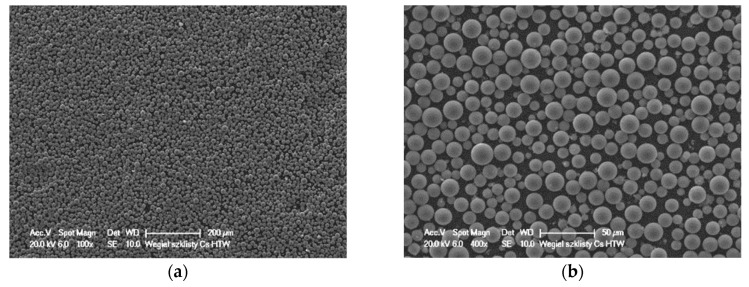
SEM images showing the morphology of spherical glassy carbon particles (GC_sf_).

Silicon carbide particles (SiC_p_) with irregular shapes and spherical glassy carbon particles (GC_sf_) were selected as aluminum matrix reinforcements. The morphology of ceramic components are shown in Figure 2 and Figure 3. Both silicon carbide and spherical glassy carbon particles were analytically pure grades with mean particle sizes in the range of 30–70 μm and 10–20 μm, respectively.

Composite suspensions with single (AlSi7Mg1Sr0.03/SiC_p_ 5 wt.%; AlSi7Mg1Sr0.03/SiC_p_ 10 wt.%; AlSi7Mg1Sr0.03/SiC_p_ 15 wt.%; AlSi7Mg1Sr0.03/GC_sf_ 5 wt.%) and hybrid reinforcements (AlSi7Mg1Sr0.03/SiC_p_ 7 wt.% + GC_sf_ 3 wt.%) were prepared by stir casting in an autoclave furnace (PTA 200/PrG) with a moving graphite stirrer system according to a previously-described procedure [7,21].

**Figure 4:**
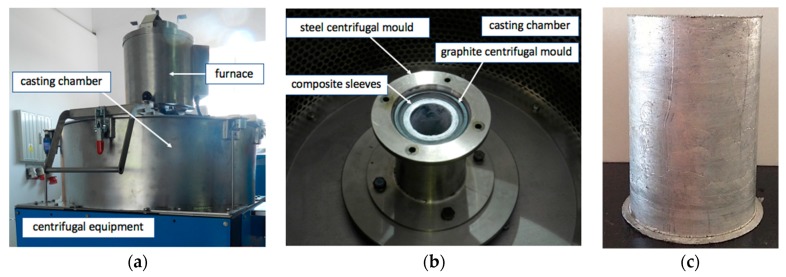
View of technological equipment (MOV500) used in the centrifugal casting process (**a**); Chamber with a visible centrifugal mold (**b**); Representative cast of composite sleeves (**c**).

The centrifugal casting process with a vertical axis was applied to shape the composite sleeves (Figure 4). Experiments were carried out using equipment (MOV500) described in a previous paper [30]. Based on experiments, model analysis [48], and preliminary tests, the centrifugal casting process parameters along the vertical axis were selected to be casting temperature (T = 720 °C), rotating mold temperature (T = 350 °C), rotating mold diameter (d = 60 mm), and mold rotational speed (3000 rpm). Light microscopy (LM) and scanning electron microscopy (SEM) were used for structural analyses.

**Figure 5:**
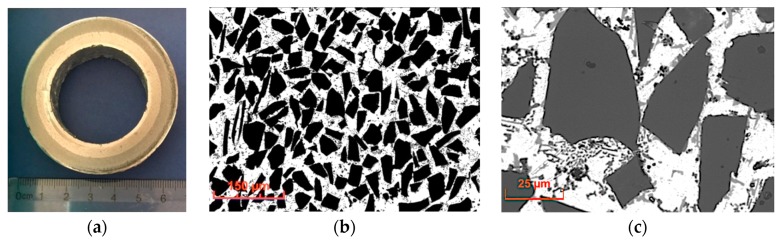
Cross-section perpendicular to the rotational axis of the centrifugal cast obtained with the AlSi7Mg1Sr0.03/SiC 15 wt.% composite suspension: (**a**) Macrostructure with visible outer composite layer; (**b**,**c**) Microstructure of the SiC_p_ particle-rich region, LM.

**Figure 6:**
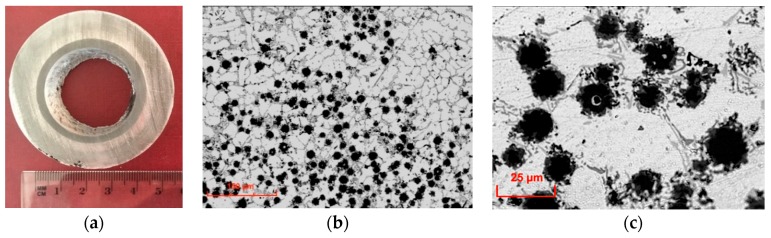
Cross-section perpendicular to the rotational axis of the centrifugal cast obtained with AlSi7Mg1Sr0.03/GC_sf_ 5 wt.% composite suspension: (**a**) Macrostructure with a visible inner composite layer; (**b**,**c**) Microstructure of GC_sf_ particle-rich region, LM.

**Figure 7:**
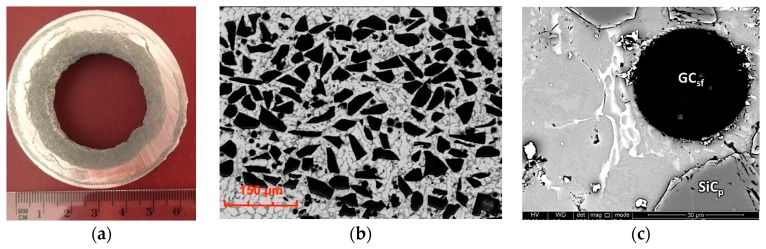
Cross-section perpendicular to the rotational axis of centrifugal cast obtained with AlSi7Mg1Sr0.03/SiC_p_ 7 wt.% + GC_sf_ 3 wt.% composite suspension: (**a**) Macrostructure with a visible internal hybrid composite layer; (**b**) Microstructure of SiC_p_ and GC_sf_ particles-rich regions, LM; (**c**) View of the boundary between the AlSi_7_ matrix alloy and SiC_p_ and GC_sf_ ceramic reinforcements, SEM.

The ceramic phases and the matrix alloy had different densities and centrifugal accelerations. This resulted in the formation of castings in the form of sleeves with single or hybrid composite layers with more reinforcing particles compared with the initial composite suspension. As shown in Figure 5, silicon carbide particles with higher densities (ρ ≈ 3.2 g/cm^3^) formed the microstructure of the outer composite layer. In turn, the lower-density glassy carbon spheres (ρ ≈ 1.4 g/cm^3^) moved toward the inner periphery and formed an internal composite layer (Figure 6). In contrast, the use of a hybrid composite suspension (AlSi7Mg2Sr0.03/SiC_p_ + GC_sf_) allowed an inner composite layer to be obtained in which the smaller glassy carbon spheres were surrounded by irregular SiC particles (Figure 7).

Tribological studies (wear resistance and coefficient of friction) were carried out in cross-sections of the composite layers formed by centrifugal casting and compared with the un-reinforced matrix alloy. Cuboid samples (Table 2) with sizes of 60 × 15 × 10 mm were cut from the composite sleeves and polished before tribological testing. Then, prepared surfaces (un-reinforced and composite layers) were subjected to abrasion tests under dry sliding conditions using a tribology pin-on-block tester [4]. A normal load of 15 N (unit pressure of 2 MPa) and a sliding speed of 0.1 m/s were used throughout the tests. The counter-pin material, Φ = 3 mm and 20 mm in length, was made of EN-GJ250 cast iron. The tests were carried out with a 9-mm stroke length over a distance of 1000 m at ambient temperature (20 °C). The obtained results were presented in the form of graphs as a function of the sliding distance.

The wear traces that appeared both on the surface of composite layers and the unreinforced matrix area were analyzed using profilometry on a MicroProf 3000, FRT optical profilometer (FRT GmbH, Bergisch Gladbach Germany). Wear trace geometry was studied immediately after the friction process. Only an ultrasonic scrubber was used to clean the surface of the tested samples. The basic surface features, such as depth of the wear trace and roughness, were assessed. 2D and 3D images were used in the analyses. The wear resistance of the tested areas was determined based on volume loss measurements of the wear traces formed on their surfaces. The research was carried out based on 3D image analysis with a 0.1 μm accuracy along the x- and y-axes, and with 0.01 μm along the z-axis.

## 3. Results and Discussion

**Figure 8:**
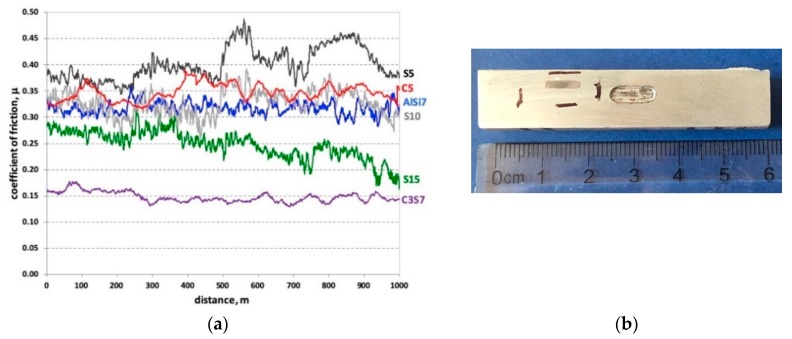
Friction coefficient (μ) versus sliding distance for un-reinforced matrix alloy (AlSi7) and different single (S5, S10, S15 and CS) and hybrid (C3S7) composite layers detailed in Table 2 (**a**); View of sample cut from the un-reinforced sleeve with a visible wear trace after tribological test (**b**).

**Figure 9:**
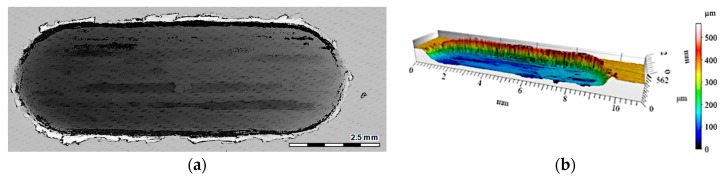
View of a wear trace in an un-reinforced area (AlSi7 matrix marked with blue line on Figure 8a) after dry sliding condition (Figure 8b): (**a**) Digital image; (**b**) Cross-section using a 3D view.

Figure 8a shows the friction coefficient measurement results as a function of the sliding distance of the AlSi7Mg2Sr0.03 matrix (AlSi7), and comparisons with areas reinforced by single (S5, S10, S15, and C5) and hybrid composite layers (C3S7). An image of a sample cut from the AlSi7 sleeve with visible wear traces after tribological tests is shown in Figure 8b. It was observed that, in the case of an un-reinforced matrix (AlSi7), the coefficient of friction erratically changes, with variations of 0.5. Such sudden changes in the friction coefficient values are characteristic of an adhesive wear mechanism, which was also confirmed by profilometry measurements of the wear track surface (Figure 9). The analyzed wear track area contained characteristic deformations at the edges with depths of 0.4 mm.

Comparing single composite layers made of suspensions containing different SiC contents (designated S5, S10, and S15) with the un-reinforced matrix, a similar trend was observed in the coefficient of friction (Figure 8a). However, its value decreased as the volume of SiC ceramic particles used in the initial composite suspension increased. The highest value of the coefficient of friction (μ = 0.4) was achieved for the S5 composite, in which the volume fraction of the reinforcing phase was 5 wt.% (black line in Figure 8a). The higher SiC content lowered the coefficient of friction. For the sample with 10 wt.% SiC particles (S10), the mean value of the coefficient of friction was μ = 0.33 (gray line in Figure 8a). The smallest coefficient of friction value (μ = 0.25), was registered for the composite with 15 wt.% reinforcing particles (green line in the Figure 8a). As can be seen, at the initial stage of friction (500 m) for the single composite layer marked S15, the coefficient of friction was significantly higher than the value recorded during the second half of the sliding distance. After the break-in period, the value of the friction coefficient during sliding changed from μ = 0.21 to μ = 0.16 at the final stage of friction. In addition, the friction coefficient value stabilized over the course of the test. The differences between the minimum and maximum values of the registered coefficient of friction ranged from 0.1 for composite S5 to 0.06 for composite S15. Based on the obtained results, it can be concluded that for the tested tribological couple, 5 wt.% SiC in the initial composite suspension did not strengthen the composite. This effect was directly related to the pull out phenomenon and the crushing of reinforcement particles during friction tests [4]. An improvement in the tribological properties was observed for 10 wt.% particles. In this case, the greater weight fraction of the ceramic particles limited the impact of the particles removed due to wear.

The nature of the friction coefficient change was significantly different for composite layers containing glassy carbon spheres. In both cases, the coefficient of friction was stable, but its value was significantly different (red and purple lines in Figure 8a). For the hybrid-reinforced composite layer marked C3S7, the value of the friction coefficient during sliding distance changed slightly from μ = 0.16 to μ = 0.14. In contrast, composite layer C5 showed a more noticeable change, and reached a value of μ = 0.35 during the final stage of friction. These results clearly indicate that the highest coefficient of friction was recorded for single composite layers S5 (μ = 0.4) and C_5_ (μ = 0.35). In turn, the use of hybrid reinforcement (SiC_p_ and GC_sf_) materials stabilized a significantly reduced the friction coefficient (mean μ = 0.15).

**Figure 10:**
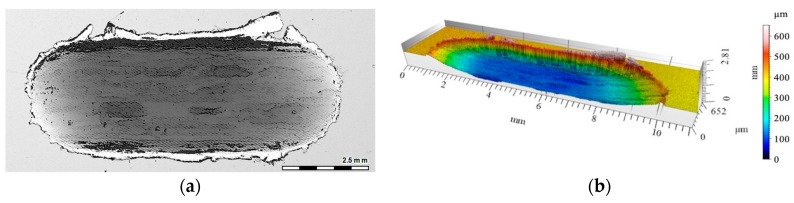
View of wear track in the S5 single composite layer (marked with a black line in Figure 8a) after dry sliding: (**a**) digital image; (**b**) cross-section through 3D view.

**Figure 11:**
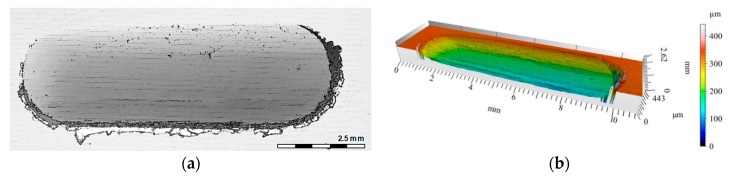
View of wear track in the S10 single composite layer (marked with a gray line in Figure 8a) after dry sliding: (**a**) Digital image; (**b**) Cross-section through 3D view.

**Figure 12:**
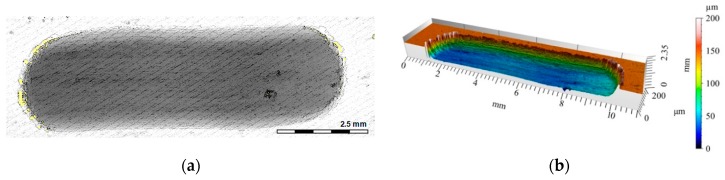
View of wear track in the S15 single composite layer (marked with green line on Figure 8a) after dry sliding: (**a**) Digital image; (**b**) Cross-section through 3D view.

The 2D and 3D images of the wear track of the single composite layers containing SiC particles are shown in Figure 10, Figure 11 and Figure 12. It was observed that for composite layer S5, the depth of the wear track was the largest at 0.6 mm (Figure 10). In turn, for the S10 composite layer (Figure 11), the depth of the abrasion mark was 0.4 mm, while it was reduced to 0.2 mm for composite S15 (Figure 12).

Furthermore, increasing the reinforcing particle content affected the nature and distribution of visible scratches on the surface of the wear trace. In the case of the S5 sample, the scratches were clearly visible, much wider, and less regular compared with scratches formed on the S10 and S15 composite surfaces (Figure 11 and Figure 12, respectively). 

**Figure 13:**
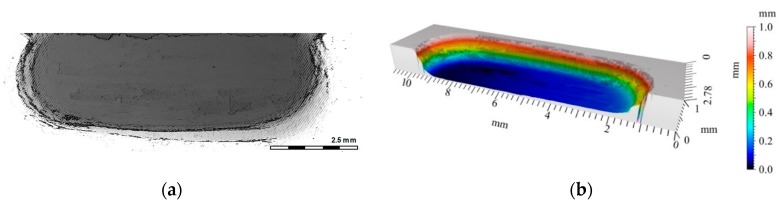
View of the wear track in the C5 single composite layer (marked with a red line in Figure 6b) after dry sliding: (**a**) Digital image; (**b**) Cross-section through 3D view.

**Figure 14:**
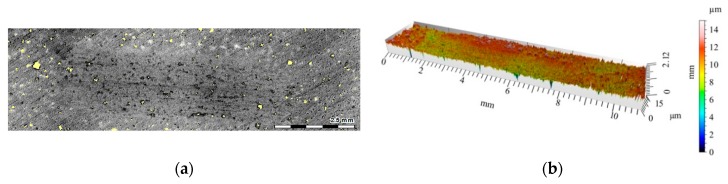
View of the wear track in the C3S7 hybrid composite layer after dry sliding: (**a**) Digital image; (**b**) Cross-section through 3D view.

Clear differences in the wear trace depth were recorded for the layers containing the carbon phases (Figure 13 and Figure 14). For the sample marked C5, the depth of the trace was 0.8 mm (Figure 13b), which was the largest among all measured values in the tribological tests. In turn, the depth of the wear trace in the C3S7 hybrid layer (Figure 14b) was significantly smaller than all other tested samples (0.1 mm). On the wear surface, fewer visible scratches were created (Figure 14a). The obtained results may be related to the specific microstructure of the hybrid composite layer (Figure 7), as well as the synergistic effects of the SiC particles which strengthened the composite layer, and the glassy carbon spheres (GC_sf_) that provided lubrication.

**Figure 15:**
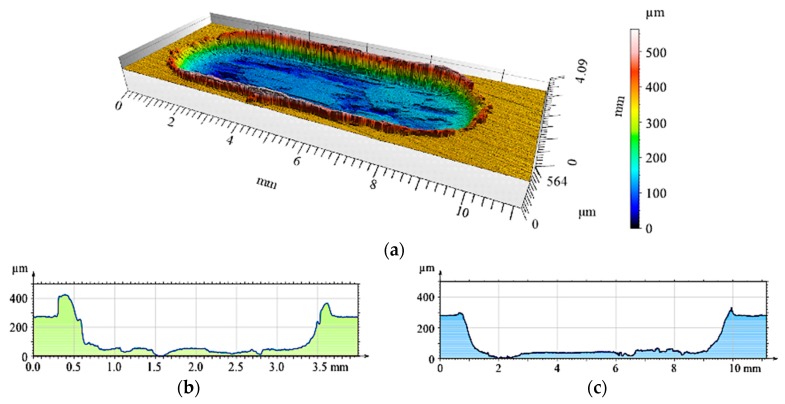
Surface geometry of the un-reinforced matrix sample (AlSi7) after working with a cast iron pin: (**a**) View of the wear track; (**b**) Roughness distribution across the friction direction; (**c**) Roughness distribution along the friction direction.

**Figure 16:**
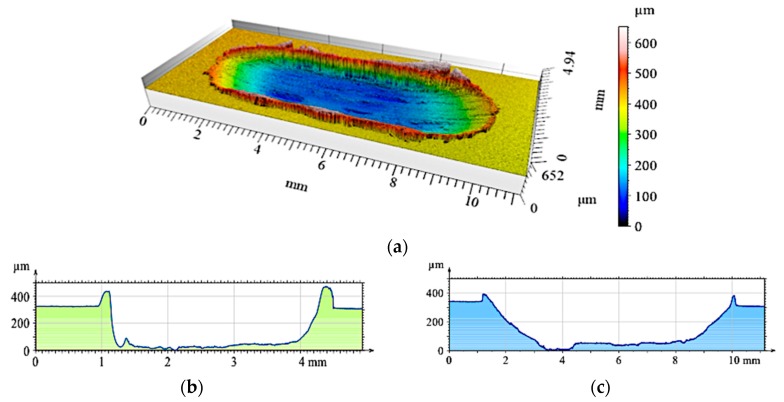
The surface geometry of the single composite layer (S5) after working with a cast iron pin: (**a**) View of the wear track; (**b**) Roughness distribution across the friction direction; (**c**) Roughness distribution along the friction direction.

**Figure 17:**
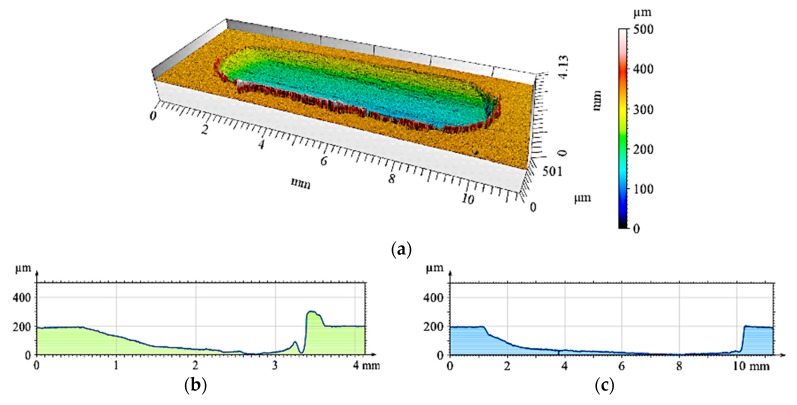
The surface geometry of the single composite layer (S10) after working with a cast iron pin: (**a**) View of the wear track; (**b**) Roughness distribution across the friction direction; (**c**) Roughness distribution along the friction direction.

**Figure 18:**
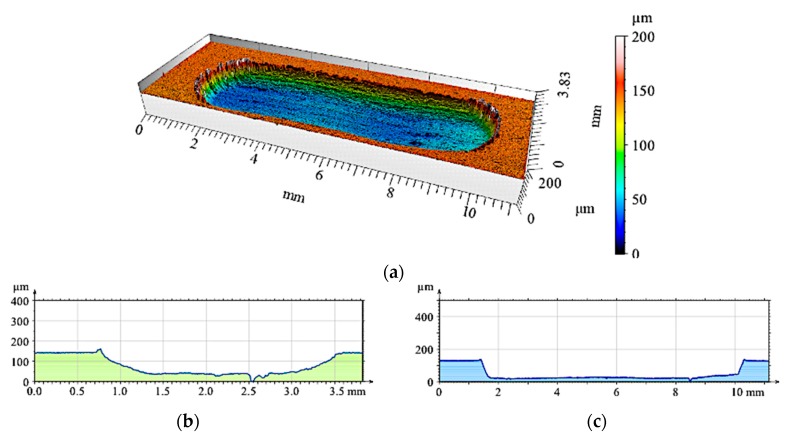
The surface geometry of the single composite layer (S15) after working with a cast iron pin: (**a**) View of the wear track; (**b**) Roughness distribution across the friction direction; (**c**) Roughness distribution along the friction direction.

**Figure 19:**
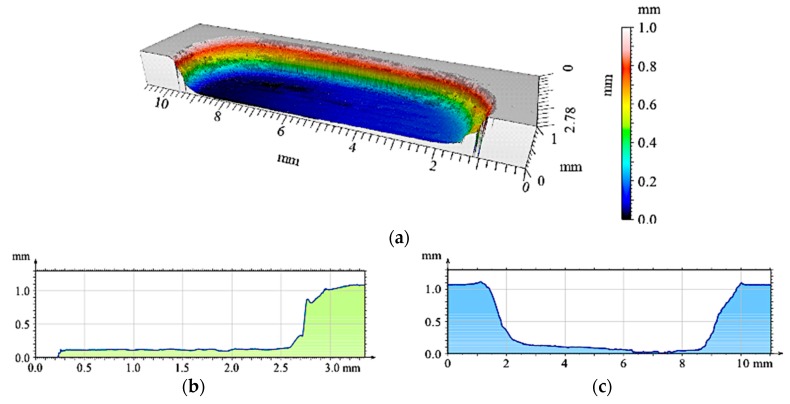
The surface geometry of the single composite layer (C5) after working with a cast iron pin: (**a**) View of the wear track; (**b**) Roughness distribution across the friction direction; (**c**) Roughness distribution along the friction direction.

**Figure 20:**
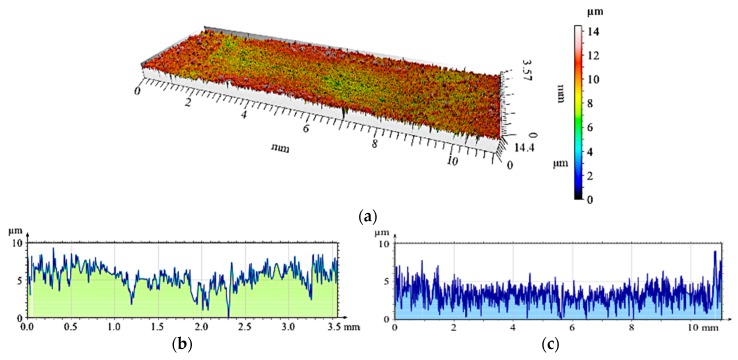
The surface geometry of the hybrid composite layer (C3S7) after working with a cast iron pin: (**a**) View of the wear track; (**b**) Roughness distribution across the friction direction; (**c**) Roughness distribution along the friction direction.

Additional profilometric tests enabled quantitative and qualitative analyses of the wear traces formed on the investigated surfaces (Figure 15, Figure 16, Figure 17, Figure 18, Figure 19 and Figure 20). The wear trace analysis of the matrix material after cooperation with the cast iron pin showed a complex surface geometry. In the bottom of the wear trace, discontinuous furrows were visible, interrupted by areas of significantly deformed material (Figure 15). Such a view of the grinding surface is characteristic of an adhesive wear mechanism. The large furrows created in the sample were closed by deformed and imposed material during friction. In the upper part of the wear trace, the material was exposed above the primary surface. The total abrasion depth was 0.6 mm, including a height above the primary surface (0.2 mm) and wear depth (0.4 mm).

In sample S5 (Figure 16), the matrix elevation at the wear track edges was lower than in the un-reinforced material, whose primary surface height did not exceed 0.2 mm. The friction depth was also reduced to 0.3 mm, but the matrix alloy still underwent plastic deformation.

Due to an increase of the SiC_p_ content in the S10 composite layer (Figure 17a), matrix deformation disappeared both in the form of elevation at the wear track edges, as well as deformations in the bottom of the friction trace observed in the S5 sample. As can be seen, the grooves were uniformly distributed and maintained their continuity over the entire friction length, but with a reduced depth. Adhesive consumption stopped, and a transition to abrasive wear was observed.

**Figure 21:**
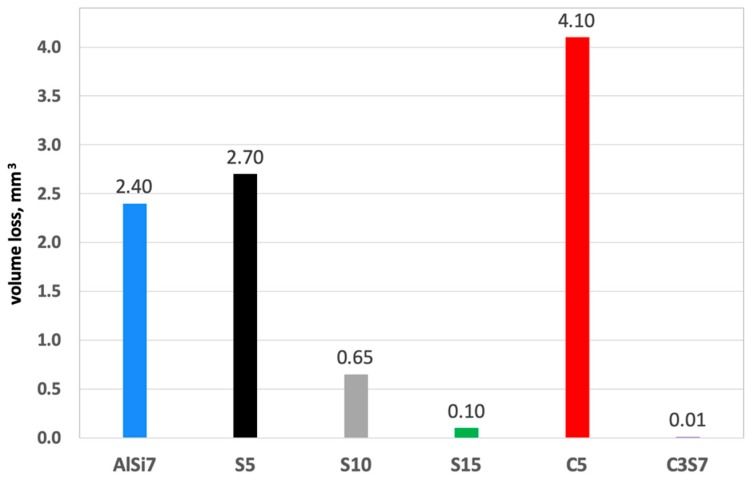
Volume loss of investigated single (S5, S10, S15 and C5) and hybrid composite layers (C3S7) compared with the un-reinforced matrix under dry sliding conditions, (accuracy of calculations 2 × 10^−8^ mm^3^).

The 3D image of the composite layer marked S15 clearly shows an abrasive wear mechanism (Figure 18a). The depth of the wear track was calculated as the maximum elevation difference of the wavy line and its lowest position. In the case of the composite layer after cooperation with a cast iron pin, the wear track depth was 100 µm (Figure 18c). The resulting abrasion was devoid of matrix elevation at the edges, and only its front part was slightly elevated. The grooves were regular and shallow, with an *Ra* value of 1.6 μm. In addition, the material volume loss was lower (0.1 mm^3^) than S5 and S10 samples (Figure 21). Thus, it was shown that increasing the amount of hard silicon carbide particles in the Al matrix significantly reduced material consumption.

In turn, the composite layer containing single vitreous carbon spheres (Figure 6) had an eight-times deeper wear track compared with SiC_p_-reinforced areas, (height difference of 0.7 mm). However, the surface roughness decreased to *Ra* = 1.3 µm, which was due to the interaction of spherical vitreous carbon on the evolution of a friction surface geometry.

A radical change in the tribological properties was obtained when using the hybrid reinforcement SiC_p_ + GC_sf_ (Figure 7). The wear track formed after cooperation with the cast iron pin (Figure 20a) was the least-visible of all tested samples. As can be observed, the vitreous carbon spheres played a decisive role in the wear process, and the surface roughness was low. In turn, silicon carbide particles improved the abrasion resistance.

The synergistic effects of the hybrid reinforcement were also confirmed by the volume loss measurements of the wear traces (Figure 21). The obtained results showed an almost 18-times smaller volume loss of the S15 single composite layer and a nearly 200-times lower value for the hybrid layer compared with the un-reinforced aluminum matrix under dry friction conditions. As can be seen, the presence of single glassy carbon spheres inside the composite layer (C5) exhibited a much smaller influence on directly reducing the abrasive wear, in terms of material volume loss. However, they play an important role as a lubricant for the working surfaces of the matrix and the SiC particles themselves (Figure 20).

For sample S5, the friction coefficient and volume loss were higher than in the un-reinforced Al matrix. This effect was characteristic of composite materials in which the properties increase after exceeding a minimum volume of reinforcement. Based on the obtained results, it can be stated that for the tested tribological couple, 5 wt.% SiC in the initial composite suspension was insufficient to obtain a strengthening effect.

## 4. Conclusions

The proper selection of technological and material parameters enabled the production of castings in the form of sleeves with a single and hybrid composite layer of different structure and tribological properties (wear resistance, friction coefficient). This is important for vertical centrifugal casting (i.e., casting temperature, rotating mold temperature, mold diameter, and speed of the centrifugal mold, as well as chemical composition of the matrix alloy, and, in particular, the size and type of reinforcement).

The investigation results showed that the friction coefficient of single-reinforced composite layers (S5, S10, and S15) decreased as the particle content in the initial suspension increased. The higher wear resistance of composite layers (S10, S15) compared with the aluminum matrix was related to the change in the wear mechanism from adhesive—abrasive to abrasive only. Moreover, the obtained results clearly showed that increasing the hard SiC particle content significantly decreased the volume loss of un-reinforced matrix under dry friction conditions. However, analyzing the surface geometry measurements, as well as the tribological characteristics the materials showed that the hybrid reinforcement provided a lower level of surface development, the lowest friction coefficient value, and thus the highest wear resistance.

Finally, it was confirmed that the use of local hybrid reinforcement in the form of silicon carbide particles combined with glassy carbon spheres significantly increased the wear resistance under dry friction conditions. The lower wear and friction, as well as the stabilization and lower friction coefficient, were the result of the synergistic effects of the hybrid reinforcement.

The presented results are part of work on shaping the functional structure of composites during centrifugal casting, and additional research is currently underway to evaluate the surface of composite layers, both before and after tribological wear. It is important to link the amount of the reinforcing phase in the composite layer to the amount of reinforcement in the suspension, as well as to microscopically assess the surface of the wear mark.

## Figures and Tables

**Table 1:** Chemical composition of EN AC 42200 (AlSi7Mg0.6) commercial alloy and matrix alloy after modification with Mg and Sr additives.

Alloy Composition	Si	Fe	Cu	Mn	Mg	Zn	Sr	Al
AlSi7Mg0.6 *	6.51	0.261	0.287	0.356	0.389	0.0665	<0.0001	92.1
AlSi7Mg2Sr0.03 **	6.21	0.272	0.265	0.356	1.43	0.0598	0.0377	91.2

* Alloy composition tested by mass spectrometry (Foundry Master). ** Alloy composition after modification by Mg and Sr tested with a mass spectrometer (Foundry Master).

**Table 2:** Designation of samples used to determine tribological properties.

Designation	Material	Volume of Particles [wt.%]	Range of Particle Size [μm]
AlSi7	AlSi7Mg2Sr0.03 matrix	-	-
S5	AlSi7Mg2Sr0.03/SiC_p_	5	30–70
S10	AlSi7Mg2Sr0.03/SiC_pB_	10	30–70
S15	AlSi7Mg2Sr0.03/SiC_p_	15	30–70
C5	AlSi7Mg2Sr0.03/GC_sf_	5	10–20
C3S7	AlSi7Mg2Sr0.03/SiC_p_ + GC_sf_	7 + 3	30–70 and 10–20, respectively
